# Connexin 43 Communication Channels in Follicular Dendritic Cell Development and in Follicular Lymphomas

**DOI:** 10.1155/2015/528098

**Published:** 2015-03-02

**Authors:** Hajnalka Rajnai, Ivett Teleki, Gergo Kiszner, Nora Meggyesházi, Peter Balla, Tamas Vancsik, Gyorgyi Muzes, Judit Csomor, Andras Matolcsy, Tibor Krenacs

**Affiliations:** ^1^1st Department of Pathology and Experimental Cancer Research, 1085 Budapest, Hungary; ^2^2nd Department of Internal Medicine, 1085 Budapest, Hungary; ^3^MTA-SE Tumor Progression Research Group, 1085 Budapest, Hungary

## Abstract

Follicular dendritic cells (FDC) show homo- and heterocellular metabolic coupling through connexin 43 (Cx43) gap junctions and support B cell selection and maturation in germinal centers. In follicular lymphomas B cells escape apoptosis while FDC develop abnormally. Here we tested Cx43 channels in reactive FDC development and follicular lymphomas. In culture, the treatment of FDC-B cell clusters (resembling to “*ex vivo*” germinal centers) with Gap27 peptide, mimicking the 2nd extracellular loop of Cx43 protein, significantly impaired FDC-B cell cluster formation and cell survival. In untreated cultures of intact clusters, cell proliferation showed a moderate reduction. In tissues, Cx43 protein levels run parallel with the density of FDC both in reactive germinal centers and in malformed follicles of follicular lymphomas and showed strong upregulation in newly generated and/or degrading bi-/multinuclear FDC of rudimentary processes. However, the inverse correlation between Cx43 expression and B cell proliferation seen in reactive germinal centers was not detected in follicular lymphomas. Furthermore, Cx43 levels were not associated with either lymphoma grade or bone marrow involvement. Our results suggest that Cx43 channels are critical in FDC and “*ex vivo*” germinal center development and in the persistence of FDC in follicular lymphomas but do not affect tumor progression.

## 1. Introduction

Follicular dendritic cell (FDC) meshwork serves as a scaffolding and antigen trap in germinal centers for boosting the generation of high affinity antibodies and B cell memory [1]. High affinity B cell clones are selected and rescued for survival through their binding antigens presented by FDC [2]. We earlier showed that, besides cell-cell adhesion and secreted cytokines, FDC can also mediate homo- and heterocellular direct interactions through cell membrane channels formed by connexin 43 (Cx43) protein [3]. Gap junction direct cell-cell communication couples members of the FDC meshwork for a functional syncytium, which can also be directly linked through Cx43 channels to germinal center B cells [3, 4]. In this study, we tested FDC-B cell cultures and follicular lymphomas (FL) of atypical FDC to see how critical Cx43 channels are in FDC development and maintenance and if Cx43 levels influence FL progression.

Gap junction channels are formed by aligning of connexin hemichannels of adjacent cell membranes [5]. The hemichannels, which may also function independently as a secretory pathway for releasing ATP, are made of six tetraspan transmembrane connexin molecules of 21 isotypes in human [6, 7]. Gap junctions allow the rapid exchange of <1.8 kDa molecules including morphogenes, second messengers (e.g., Ca^2+^, IP_3_, and c-AMP), metabolites (e.g., nucleic acids and amino acids), and linear antigenic peptides of <1.2–1.8 kDa between cells at selective permeability depending on the isotype(s) involved [8]. Connexins and their channels can mediate signals concerned with cell differentiation, apoptosis, cell cycle control, and the formation of functional compartments within tissues [9–11]. Connexins are expressed in most immune cells and have been linked to the regulation of early hematopoiesis and immunoglobulin production and to the promotion of cellular immune response through antigen cross-presentation [5, 12].

FDC is also an important component of FL, an indolent neoplasia of follicular B cells, which frequently show t(14;18)(q32;q21) chromosomal translocation resulting in the overexpression of the antiapoptotic Bcl-2 protein [13, 14]. FL tumor cells, characterized by CD10, bcl-2 and bcl-6 immunoreactions, are arranged in follicular structures of irregular sizes that may resemble reactive follicles but with a lost polarization [15, 16]. Follicular areas can be highlighted by CD21, CD23, and CD35 positive distorted FDC meshwork which shows alternating focal hyperplasia and fragmentation [17]. Diminishing and lost follicular pattern in grade 3 FL correlates with the progression of FL into diffuse large B cell lymphoma (DLBCL) of poor outcome in 25–35% of the patients [14]. Bone marrow involvement of FL affecting 40–70% of the cases, where FDC and follicular structures may also form, indicates an adverse FL prognosis too [18–20]. On the other hand, stromal cells including FDC can support lymphoma B-cell survival and may contribute to the resistance both to chemotherapy and biological (trastuzumab) therapy targeting CD20 protein in tumor cells with a humanized monoclonal antibody [21–24].

In cultured low density cell fractions of reactive human tonsils, FDC-B cell clusters can form resembling to* ex vivo *germinal centers [4, 25]. In this study, Cx43 docking and channel functions were perturbed by treating of these cultures with Gap27 connexin mimetic peptide which corresponds to the second extracellular loop (E2) of Cx43 protein [26]. Gap27 peptide significantly impaired the formation of FDC-B cell clusters and cell survival. Cx43 expression showed strong statistical correlation with the FDC meshwork both in reactive germinal centers and in FL, and it was upregulated in bi- or multinuclear FDC of underdeveloped processes. However, Cx43 levels had no significant association with either neoplastic B cell proliferation, tumor grade, or bone marrow involvement in FL. Our results suggest that Cx43 channels are critical in FDC and in “*ex vivo*” germinal center development and in the persistence of FDC in FL but may not play a significant role in FL progression. Since FDCs nurse FL B cells, inhibition of Cx43 in FL would likely to hinder FDC growth and eliminate its trophic factors for tumor cell survival and support chemotherapy and/or biological therapy.

## 2. Materials and Methods

### 2.1. FDC-B Lymphocyte Cultures

Low density cell fractions of human tonsils, known to be enriched in activated germinal center B lymphocytes and FDC, were isolated with the consent of the patients as described before [4, 27]. All reagents specified were from Sigma-Aldrich (St Louis, MO). Briefly, surgically excised tonsils were minced and then digested twice for 30 min each in 200 U/mL collagenase IV (C5138) and 10 U/mL DNase I (D4263) containing 90 *µ*g/mL gentamycin (G1272) in 30 mL Iscove's modified Dulbecco's medium (IMDM; I3390), at 37°C. The supernatants kept on ice were centrifuged at 600 g for 10 min, resuspended in IMDM, and layered (at 1 ×g) on discontinuous BSA gradients consisting of layers of 1.5%, 2.5%, and 5% BSA (A9706) in Hanks' balanced salt solution (H6648). Then the low density cells between the interfaces of 2.5% and 5% BSA were layered on Percoll gradients (P4937) and centrifuged at 1200 ×g at 4°C for 20 min to allow collection of <1060 mg/mL density cells. Isolated cells were either cytospinned or cultured on 24 × 24 mm microscope coverslips (2–4 × l0^5^ cells) for 2–24 h in IMDM containing 0.1% gentamycin and 10% fetal calf serum under 5% O_2_, 5% CO_2_, and 90% N_2_. For immunostaining, cells were fixed in acetone for 10 min and dried at room temperature for 30 min.

### 2.2. Blocking of Cx43 Gap Junctions with Gap27 E2 Domain Mimetic Peptide

After preliminary testing, Gap27 connexin mimetic peptide (G1794; Sigma-Aldrich) mimicking the aa204–214 sequence on the 2nd extracellular loop of Cx43 (SRPTEKTIFII) was added to the culture media in 200 *μ*M final concentration onto each coverslip cultures containing 2–4 × 10^5^ cells isolated from reactive human tonsils. Cell phenotype, all cell numbers, and average cell numbers within clusters were monitored and compared between Gap27 treated cultures and cultures treated with either 100 *µ*g BSA (untreated) or a scrambled peptide sequence (TFEPDRISITK) at 2 , 4, 6, 12, 16, and 24 h in at least 3 parallel coverslip cultures each time by counting 300 cells on each coverslip. Coverslip cultures were either immunostained (see below) or analyzed after nuclear staining with 7-aminoactinomycin D (7-AAD; 1 : 1000; Invitrogen, Carlsbad, CA) for 1 min, postfixed in 4% neutral buffered formaldehyde for 10 min, and mounted using Faramount (Dako, Glostrup, Denmark). Samples were tested with Leica TCS4D confocal laser scanning microscope (Leica Lasertechnique, Heidelberg, Germany) using single and multichannel fluorescence combined with differential interference (Nomarski) optics.

### 2.3. Biopsy Samples and Tissue Microarray

Formaldehyde-fixed, paraffin embedded lymph node biopsies of 35 untreated FL patients and the corresponding bone marrow core biopsies from 20 patients were selected for this study from the archives of the 1st Department of Pathology and Experimental Cancer Research (Budapest). The diagnoses were based on standard criteria using the classification of the World Health Organization (WHO) by considering histopathology, immunophenotype, and molecular features [14]. Clinicopathological data of the studied patients are summarized in Table 1. Bone marrow sampling took place within 2 weeks of the primary diagnosis of FL. Archived, reactive lymph node samples from 3 patients were used as normal controls. Representative areas were selected from each paraffin block based on routine hematoxylin and eosin staining and at least duplicate cores of 2 mm diameter were collected into tissue microarray (TMA) blocks using the computer-assisted TMA Master (3DHISTECH Ltd, Budapest, Hungary) [28]. The use of all human tissues including native tonsils for research purposes in this study has been approved by the Ethical Board of Semmelweis University (TUKEB 7/2006).

### 2.4. Immunohistochemistry

Four *μ*m thick TMA sections were cut, mounted on charged adhesive slides (SuperFrost Ultra Plus, Braunschweig, Germany), and immunostained following routine dewaxing. For heat induced epitope retrieval, in an electric cooker at ~105°C for 28 min (Awair, Biofa Kft, Veszprem, Hungary), a mixture of 0.1 M Tris-base and 0.01 M EDTA, pH 9.0, was used, except for detecting CD21 where a pH 6.1 target retrieval solution (TRS, Dako) was applied. After blocking the endogenous peroxidases in 1% H_2_O_2_ for 20 min the sections were incubated with primary antibodies (all from Dako, if not otherwise noted) including mouse monoclonal anti-CD10 (clone: 56C6, 1 : 20), anti-CD21 (clone: 1F8, 1 : 20), anti-CD23 (clone: 1B12, 1 : 10, Leica-NovoCastra, Newcastle upon Tyne, UK), anti-low-affinity nerve growth factor receptor (LNGFR, clone: 7F10, 1 : 300; AbCam, Cambridge, MA), and Ki67 (clone: Mib1, 1 : 50) IgG at room temperature, for 60 min. Tissue-bond antibodies were detected using the NovoLink (RE7140-CE; Leica) polymer peroxidase system by incubating the slides with the postprimary reagent for 30 min and then with the peroxidase polymer conjugate for 40 min. Peroxidase activity was revealed with the diaminobenzidine-H_2_O_2_ chromogen-substrate system for 5–8 min. After each incubation step the slides were washed in 0.1 M Tris-buffered saline pH 7.4 (TBS) including 0.05% Tween 20 for 3 × 3 min and, finally, were counterstained with hematoxylin.

### 2.5. Immunofluorescence and Double Staining

After fixation in methanol-acetone (1 : 1) for 10 min, FDC-B cell cultures grown on microscope coverslips were also tested with immunofluorescence using rabbit anti-Cx43 (code: #3512, 1 : 200; Cell Signaling, Beverly, MA) and mouse anti-Ki67 (clone: Mib1, 1 : 100) IgG or combining either monoclonal mouse anti-Cx43 (Clone: CX-1B1, 1 : 100, Life Tech., Grand Island, NY) with rabbit anti-human IgM (A0425, 1 : 300), or IgG (A0423, 1 : 500) antibodies or rabbit anti-Cx43 (code: #3512, 1 : 200) with mouse anti-CD35 (clone: Ber-Mac-DRC, 1 : 50) antibodies for double labeling. Mouse and rabbit antibodies were detected with either Alexa488 (green) or Alexa546 (orange-red) labeled anti-mouse or anti-rabbit IgG, diluted in 1 : 200 (all from Invitrogen-Molecular Probes, Carlsbad, CA). Immunofluorescence was also used on FL TMA paraffin sections, following the same section pretreatment described above. Sections were incubated with rabbit anti-Cx43 (#3512, 1 : 100) or monoclonal mouse anti-Cx43 (clone: CX-1B1, 1 : 100) IgG either alone or in relevant combinations (mouse and rabbit) with rabbit anti-desmoplakin IgG (Dp12, 1 : 200; generous gift from A.I. Magee, National Institute for Medical Research, Mill Hill, London, UK), monoclonal mouse anti-CD4 (clone: 4B12, 1 : 20), anti-CD10 (clone: 56C6, 1 : 30), anti-CD21 (clone: 1F8, 1 : 20), or Ki67 (clone: Mib1, 1 : 100) IgG at room temperature, overnight using the same immunofluorescence detection reagents as for cultured cells. Cell nuclei were stained with either Hoescht (blue) or 7-aminoactinomycin (red). The slides were finally mounted with a gelatin-based medium Faramount.

### 2.6. Scoring and Automated Image Analysis of Signals

In FDC-B cell cultures, the number of cells within clusters, the number of all cells (indicating cell survival), and Ki67 positive cells (and proliferating cell fractions) was determined in 3–5 representative, calibrated ×40 magnified images of 3 parallel samples at each time point. For all cells and Ki67 positive cells the findings were extrapolated for 1 mm^2^. Immunostained TMA slides were subjected to whole slide digitalization at either bright-field or fluorescence using Pannoramic Scanner (3DHISTECH) equipped with a high resolution ×20 objective (NA = 0.83; Carl Zeiss, Jena, Germany). Representative CD10 positive FL areas were scored independently by two experts (HR and TK) for Cx43, CD21, CD23, and Ki67 by assessing the proportion (%) of tumor cells positive for the given biomarker using a 4-tier scale where score 0 represented <10%; score 1 represented 10–25%; score 2 represented 26–50%; and score 3 > 50% of Cx43 positive area fraction of the tumors, and the results were consolidated based on agreement. For further analysis Cx43 and Ki67 immunopositive area fractions in % were determined in CD10^+^/CD21^+/high^ and CD10^+^/CD21^−/low^ areas in reactive follicles, FL lymph nodes, and in bone marrow involvement of FL using standard thresholds in the HistoQuant software (3DHISTECH).

### 2.7. Statistics

Statistical analysis was performed using the SPSS 15.0 software package (IBM-SPSS Inc., Chicago, IL). Spearman's rank test was used for revealing correlations between biomarker levels. Statistical significance between rank variable scores of CD21 (FDC) and Cx43 reactions in CD10 positive FL areas and bone marrow involvement of FL was analyzed using the Fisher exact test after dichotomization of scoring data at the most reproducible threshold 0-1 (low) versus scores 2-3 (high). The Wilcoxon paired nonparametric test was used to compare variables in FDC-B cell culture experiments and for testing Cx43 and Ki67 protein levels in CD10^+^/CD21^+^(high) and CD10^+^/CD21^−^(low) FL areas. *P* values of <0.05 were considered as statistically significant.

## 3. Results

### 3.1. Cx43 Expression in Reactive Lymphoid Follicles

**Figure 1:**
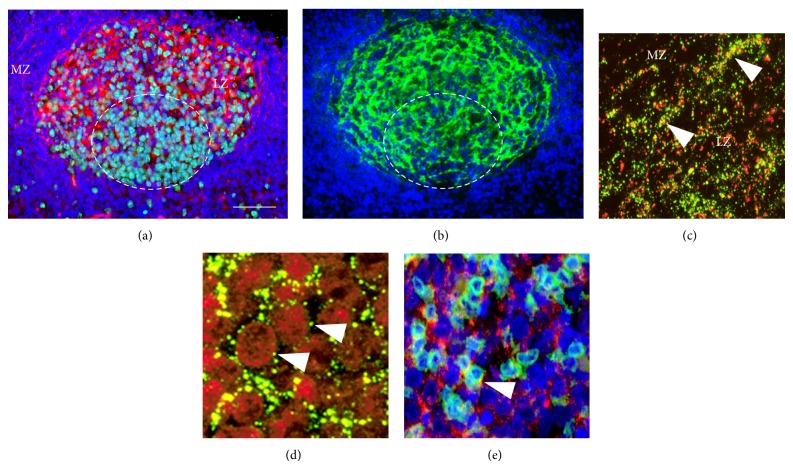
Connexin 43 expression in secondary lymphoid follicles of reactive human tonsils. Cx43 immunoreaction (red) is accumulated mainly in the light zone localizing less Ki67 positive lymphocytes ((a), green) and more CD21 positive FDC processes ((b), green) than the dark zone (circled areas) of germinal center in consecutive sections. Cx43 (green) colocalizes with desmoplakin (red) produced by FDC ((c); arrowheads). Cx43 plaques (green) are also closely associated with B cells ((d); arrowheads) and rarely with CD4 positive T cells ((e); Cx43: red, CD4: green, arrowhead) in the germinal center. Immunofluorescence, nuclear staining in (a), (b), and (e) with Hoescht (blue) and in (d) with 7-aminoactinomycin D (red). LZ: light zone and MZ: mantle zone. Scale bar on (a) shows 30 *µ*m on (a) and (b); 15 *µ*m on (c) and (e); and 7 *µ*m on (d).

In reactive lymphoid follicles particulate Cx43 immunoreaction was primarily associated to the FDC meshwork identified with CD21 (C3d, complement receptor) immunostaining (Figures 1(a)-1(b)). Significantly higher Cx43 levels were seen in the light zone where FDC was more developed and proliferating B cells were fewer than in the dark zone (see also Figures 4(l) and 4(m)). Cx43 reaction colocalized with CD21 and the desmosomal adhesion protein desmoplakin, both produced by FDC (Figure 1(c)). It was also detected in B cells (Figure 1(d)) and less frequently in CD4 positive T cells (Figure 1(e)) within germinal centers.

### 3.2. Blocking of Cx43 in FDC-B Cell Cultures,* Ex Vivo* Germinal Centers

**Figure 2:**
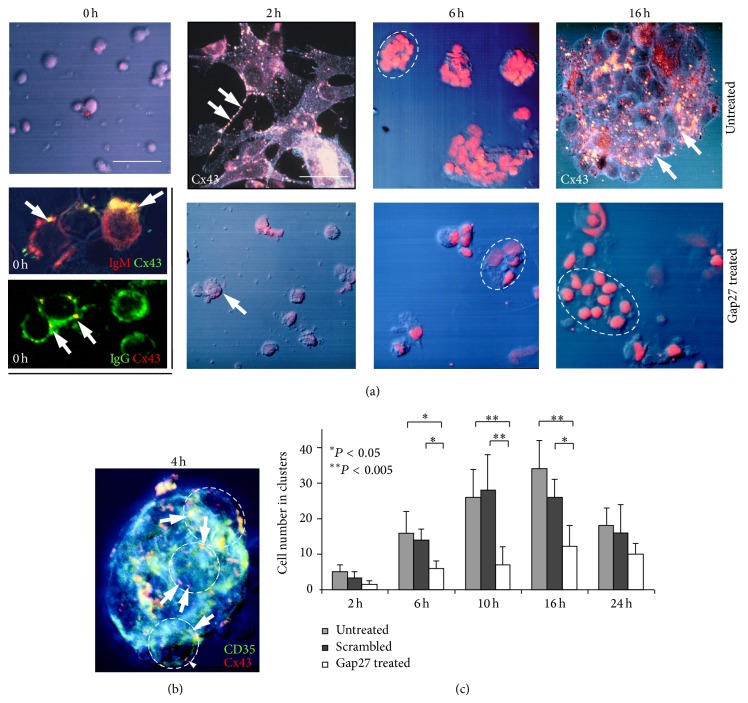
Treatment of low density cell fractions of reactive human tonsils in culture using 200 *µ*M Gap27 connexin mimetic peptide. (a) At the start (0 h) round cells are dispersed, some of them coexpressing Cx43 and either IgG or IgM (arrows). In control (untreated) cultures (1st row) at 2 h, FDC processes decorated with Cx43 (arrows) enmesh round B cells. By 6 h clusters are formed of 10–15 FDC and B cells which grow up to ~50 cells by 16 h, with Cx43 plaques detected at the cell borders (arrows). In Gap27 treated cultures (2nd row) the cluster formation is seriously compromised resulting in cells of vacuolated cytoplasm (arrow) and shrunken nuclei. (b) A “healthy” FDC-B cell cluster of ~8–10 cells (some are encircled) with Cx43 particles (red) colocalizing in B cell membranes (yellow, arrows) with sheets of CD35 positive FDC (green), which envelop them. Immunofluorescence single and double labeling and Nomarski differential interference combined with nuclear staining using either 7-aminoactinomycin D ((a), red) or Hoescht ((b), blue). (c) Graph showing significantly (^*^ *P* < 0.05; ^**^ *P* < 0.005) reduced cell numbers within clusters after Gap27 treatment compared either to untreated or scrambled-probe treated cultures. Results in graphs show the mean and standard deviation of at least three independent experiments. Scale bar shows 50 *µ*m, except in the untreated Cx43 labeled cultures (upper row) where it is 25 *µ*m. In the left row middle panels it equals 15 *µ*m and 10 *µ*m in the bottom panel.

Isolated low density tonsillar cells enriched in activated B lymphocytes and FDC formed clusters in 2–24 h cultures, which mimicked developing germinal centers* ex vivo* including gradually growing numbers of B cells enveloped by protruding sheets of a few FDC. The main features of this process and the results of treating these cultures with Gap27 peptide of identical sequence with the 2nd extracellular domain of Cx43 protein are summarized in Figure 2. Freshly isolated round cells expressing IgM, IgG, or rarely IgD were decorated with a few Cx43 plaques in their membranes and were accompanied by a few CD35 positive presumed FDC (Figure 2(a)). Rare CD4^+^ T lymphocytes were also seen but without clear Cx43 positivity. In untreated 2 h cultures, FDC processes projecting towards and embracing B cells were densely decorated with Cx43 plaques. By 4 h, clusters made up of 8–10 cells were formed where Cx43 in B cell borders colocalized with the CD35 reaction of FDC (Figure 2(b)). As estimated with double labelling, each FDC interacted with 3–5 B lymphocytes within cell clusters. From 6 h on, gradually increasing numbers of cells were involved in clusters reaching >50 cells/cluster by 16 h. The average number of cells involved in clusters between 2–16 h was significantly reduced and FDC processes were underdeveloped after Gap27 treatment compared to both the untreated and the indifferent (scrambled) peptide treated cultures (Figure 2(c)). Furthermore, in Gap27 treated cultures, elevated numbers of damaged cells showing vacuolated cytoplasm and nuclear shrinkage suggestive of programmed cells death were seen.

**Figure 3:**
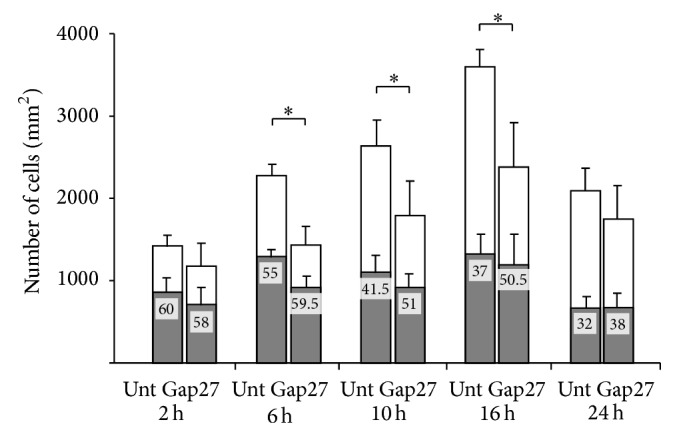
Testing of cell proliferation (gray columns) and proliferating cell fractions (numbers in gray columns in %) using Ki67 immunocytochemistry and absolute cell numbers (gray + white columns) indicating cell survival, in FDC-B cell cultures. Absolute cell numbers are significantly decreased (^*^ *P* < 0.05) 6 h, 10 h, and 16 h after Gap27 treatment compared to the untreated cultures (Unt). Proliferating cell fractions show a nonsignificant trend of reduction at these time points (*p*_6 h_ = 0.423; *p*_10 h_ = 0.186; *p*_16 h_ = 0.067) in the control cultures. Results in graphs show the mean and standard deviation of at least three independent experiments.

There was a significant decrease in the absolute cell numbers in the Gap27 treated compared to the untreated cultures (*P* < 0.05) at 6 h, 10 h, and 16 h (Figure 3). Though the absolute number of Ki67 positive cells did not differ much, proliferating cell fractions showed a nonsignificant trend of reduction in untreated compared to Gap27 treated cultures, except at 2 h.

### 3.3. Cx43 Expression in Follicular Lymphomas

**Figure 4:**
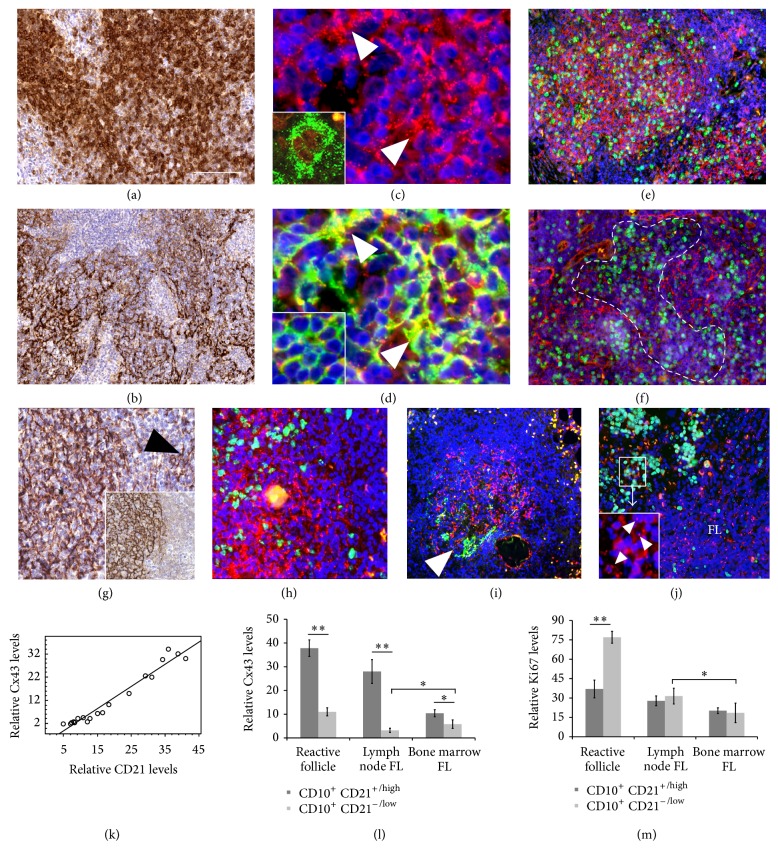
Connexin 43 expression in follicular lymphomas (FL); lymph node localization ((a)–(f)); and bone marrow involvement ((g)–(j)). Large and irregular follicles dominated by CD10 positive FL B cells (a) and fragmented CD21 positive FDC meshwork (b). Cx43 ((c), red) reaction colocalizes with that of CD21 ((d), green) in yellow (arrowheads). Insets show a binuclear FDC with highly upregulated Cx43 ((c), green),and colocalization of Cx43 (red) and CD10 (green) as yellow dots (d). Elevated tumor cell proliferation detected with Ki67 reaction (green) is seen in FL follicles either with dense FDC and Cx43 reactions ((e); red) or with fragmented FDC and Cx43 staining ((f); circled area highlights broken or missing FDC). In the bone marrow involvement of FL NGFR (g) and CD21 (inset) positive follicles (arrowhead: LNGFR positive hyperplastic stromal cells) show the most of Cx43 reaction ((h), red) and proliferating tumor cells (Ki67-green). Strong Cx43 staining ((i), red) is accompanied with only fragmented CD21 reaction (green) in another follicle. Significantly more Cx43 (red) but less Ki67 (green) is detected within a bone marrow FL infiltrate than in the rest of bone marrow (j). Arrowheads in the inset show Cx43 within the boxed area. Graphs reveal linear correlation between Cx43 and CD21 levels in FL (k) and Cx43 (l) or Ki67 (m) expression in FDC rich and FDC poor FL areas. Immunoperoxidase ((a), (b), and (g)) and immunofluorescence staining ((c)–(f) and (h)–(j)). Blue nuclear staining using hematoxylin ((a), (b), and (g)) or Hoescht ((c)–(f) and (h)–(j)). Significance: ^*^ *P* < 0.05 and ^**^ *P* < 0.005. Scale bar on (a) shows 150 *µ*m on (a) and (b); 30 *µ*m on (c) and (d); 120 *µ*m on (e), (f), (g), (i), and (j); and 100 *µ*m on (h).

CD10 positive FL follicles lost polarization compared to reactive germinal centers and frequently enclosed a fragmented FDC meshwork detected with CD21 immunoreaction (Figures 4(a)-4(b)). Similar to that in reactive follicles, Cx43 expression was significantly associated with the FDC meshwork (Figures 4(c)-4(d)), while Cx43 levels were very low in CD10 positive but CD21 negative/low tumor areas. Cx43 protein was highly upregulated in bi- or multinuclear FDCs with rudimentary processes and was partly colocalized with CD10 protein on FL B cells. In tumor regions without obvious vessels, reactive centroblasts, or extracellular matrix, cell proliferation was not significantly different between areas of compact or fragmented CD21/Cx43 double positive FDC (Figures 4(e)-4(f)). In bone marrow FL infiltrates, the highest Cx43 levels were detected in CD21/NGFR positive follicles (Figures 4(g)-4(h)), where Cx43 levels were usually lower and FDC was more fragmented (Figure 4(i)) than in lymph node FL follicles. Furthermore, Cx43 levels were higher in FDC-free diffuse bone marrow FL infiltrates than either in the preexisting bone marrow (Figure 4(j)) or in the diffuse FDC-free FL areas in lymph nodes. On the contrary, proliferating cell fractions were significantly higher in the preexisting marrow than in bone marrow FL infiltrates. Both FDC and stromal cell processes were positive for NGFR and occasionally showed continuity with each other in FL infiltrates at both localizations. Few Cx43 signals were also seen in NGFR positive stromal cell processes. Graphs summarize the significant association between CD21 and Cx43 levels in FL samples (Figure 4(k)) and the correlations of Cx43 or Ki67 levels with CD10^+^/CD21^+^(high) and CD10^+^/CD21^−^(low) FL areas (Figures 4(l)-4(m)). Spearman rank correlation was also highly significant between Cx43 and CD21 expression (rho = 0.981, *P* < 0.001), supporting the colocalization of these proteins. This test showed only a weak negative trend either between Cx43 and Ki67 (rho = −0.154) or between CD21 and Ki67 (rho = −0.128) expression.

In lymph node FL, there was no significant correlation between Cx43 expression and bone marrow involvement or tumor grade (Table 2) and between the proportion of FDC, detected using CD21 or CD23 immunoreactions, and tumor grade (*p*_CD21_ = 0.449; and *p*_CD23_ = 0.112).

## 4. Discussion

Using cultured FDC-B cell clusters, we earlier revealed that Cx43 gap junctions functionally couple cells of the FDC meshwork and FDC to germinal center B cells [4]. Here we show in the same model that interfering with Cx43 docking and channel functions, by using Gap27 connexin mimetic peptide, can significantly impair the development of FDC-B cell clusters and cell survival. In tissues, Cx43 protein expression runs parallel to the density of FDC both in reactive germinal centers and in disorganized FL follicles. It is highly upregulated in large FDC bodies of rudimentary processes, consistent with either newly developing or degrading FDC. In reactive germinal centers, local Cx43 levels show an inverse correlation with B cell proliferation, which is lost in FL. Still Cx43 expression in FL lymph nodes is not linked statistically to either FL grade or bone marrow involvement. These data suggest that Cx43 protein and its channels are important in FDC meshwork and in “*ex vivo*” germinal center development and in the persistence of FDC in FL but do not influence significantly FL progression.

Cx43 is the most ancient and prevalent connexin isotype, in which involvement in immune functions has been reported by several groups [12]. In the humoral arm, blocking of Cx channels was shown to inhibit the immunoglobulin production of B cells [29]. Cx43 gap junctions were also implicated in the LFA1-induced Rap1 GTPase activation, which can mediate CXCL12-directed B cell spreading, migration, and adhesion [30, 31], which are important in germinal center reaction. Cx43 channels have also been involved in the regulation of T cell maturation and proliferation, in particular of CD4/Foxp3 double positive regulatory T cells [32–34] and in the cross-presentation of tumor or viral peptides between cells [35–37].

The contribution of Cx43 in FDC development and germinal center reaction was raised earlier by our detection of progressive accumulation of Cx43 plaques during the formation of FDC and secondary lymphoid follicles after repeated antigen challenge in mice [4]. In line with this, CXCL13, an essential chemokine for FDC and lymph node generation, can be induced by retinoic acid derived from vitamin A, a known promoter of Cx43 channels [38–40]. Here we provide further evidence on the impact of Cx43 in FDC growth and potential involvement in germinal center functions. Binding of Gap27 to the 2nd extracellular loop of Cx43 hemichannels is known to result in early channel closure and diminishing cell coupling [41]. Since FDCs normally grow long processes and establish strong interactions with each other, which look abortive and disrupted in Gap27 treated cultures, FDC adherence may also be affected through Gap27 directly preventing anchorage of the opposing connexin loops.

In germinal centers, FDC provide a supportive niche for B cell survival involving direct FDC-B cell interactions including CXCL13/CXCR5, CXCL12/CXCR4, ICAM-1/LFA-1, and VCAM-/VLA-4 antigen trapped by CD21 and CD35 complement receptors or by FC*γ*R and FC*ε*R (CD23) immunoglobulin Fc receptors for reaching out by B cell receptors as well as by releasing cytokines such as BAFF, IL-6, and IL-15 (16) [1, 42–45]. In addition, Cx43 channels are formed by both FDC and B lymphocytes, which permit their homo- and heterocellular (FDC-B cell) metabolic coupling [3, 4, 46, 47]. In culture, activated B cells are also prone to apoptotic cell death without FDC help and/or T cell interactions [48]. By interfering with FDC development using Gap27 peptide, FDC support of B cell survival is also abused, resulting in concomitant damage of B cells. Besides reducing cell numbers within clusters, Gap27 treatment also led to a significant decrease in the absolute cell numbers, while proliferating cell fractions were moderately elevated compared to untreated controls. This is consistent with a significantly reduced cell survival and increased detachment and loss of damaged cells after Gap27 treatment, while numerous cells which are dominantly out of clusters can still proliferate. It is likely that both gap junctions and hemichannels are affected and both homo- and heterocellular Cx43 channels which are involved [41]. However, further studies are required to clarify the mechanisms of action of Gap27 peptide on FDC-B cell interactions, B cell maturation, and the potential involvement of hemichannels.

In FL B cells, forced Bcl-2 expression protects tumor cells from apoptosis, but B cell survival factors released by FDC and T cells are still important for FL B cells persistence. In line with this, FL B cells cannot be grown in culture without factors produced by FDC and CD4^+^ T helper cells including CD40L, IL-2, and IL-4 [49–51]. Since Cx43 channels may support FDC development and survival even in FL, blocking of Cx43 would most likely impair FDC and its production of B cell survival factors, which have been implicated in the FDC mediated therapy resistance of FL B cells [22–24].

The FDC meshwork is frequently distorted in FL, showing local hyperplastic cell bodies with rudimentary processes and complete loss of FDC in CD10^+^ diffuse tumor infiltrates [14]. Since normal germinal center structure is based on trophic and regulatory factors shared between T helper cells, B cells, and FDC, deformed FDC reflects inappropriate trophic signaling by malignant B cells [52]. The importance of Cx43 channels in syncytial FDC is reflected by their following of the pattern and density of FDC detected with CD21 (and CD23) immunoreactions both in reactive germinal centers and in FL follicles. In addition, Cx43 protein is particularly upregulated in bi- or multinuclear FDCs of large cell bodies but rudimentary processes, which are likely to be either newly forming or degrading FDC in FL. These findings are in line with our results in Gap27 treated cultures by both supporting the role for Cx43 channels in FDC development and survival.

Cx43 channels have been implicated in cell cycle control and cell differentiation [53]. In reactive germinal centers the inverse correlation between Cx43 expression and proliferating B cell fraction suggested Cx43 a role in the control of B cell proliferation. A similar but nonsignificant trend was observed in untreated FDC-B cell cultures of intact cell clusters. Also, in the diffuse bone marrow infiltrates of FL, LNGFR positive stromal cell hyperplasia was accompanied by significantly higher Cx43 expression, lower B cell proliferation, and tumor grade. It is of note, however, that tumor microenvironment including elevated CD8^+^ T cells, forkhead box protein 3 (FoxP3)^+^ T cells, and CD68^+^ macrophages can also contribute to the downgrading of bone marrow FL [28]. Nevertheless, Cx43 levels in lymph node FL showed no significant link with bone marrow involvement or tumor grade, two known indicators of FL progression.

In conclusion, the exact role of Cx43 channels and cell coupling in germinal center is still needed to be clarified. Our earlier study showed that the FDCs are coupled through Cx43 gap junctions and Cx43 protein is upregulated in FDC during repeated immune challenge [4]. Here we revealed that Cx43 channels are crucial for FDC to develop into a syncytial network which support the survival of FDC-B cell clusters and for the persistence of FDC in FL but do not influence significantly the progression of FL. Since both B cells and T cells can produce Cx43 channels and are in direct contact with the functionally coupled FDC meshwork, it is likely that Cx43 channels are involved in the integration of germinal center functions. Details of the potential metabolic cooperation between FDC, B cells, and T cells need further* in vivo* studies, which have so far been prevented by the complexity germinal center reaction.

## Figures and Tables

**Table 1:** Clinicopathological data of follicular lymphomas studied.

		Bone marrow involvement		Total
		Presence	Absence	

Sex	Female	12 (60%)	9 (60%)	21 (60%)
	Male	8 (40%)	6 (40%)	14 (40%)

Age at diagnosis	Median	48.65	49.67	49.16
	Range	26–82	33–74	26–82

Grade	1	1 (5%)	3 (20%)	5 (12.5%)
	2	16 (80%)	6 (40%)	24 (60%)
	3	3 (15%)	6 (40%)	11 (27.5%)

Histologic pattern	Follicular	20 (100%)	12 (85%)	32 (92.5%)
	Diffuse	0 (0%)	3 (15%)	3 (7.5%)

FLIPI	Low	2	9	11
	Intermediate	12	5	17
	High	6	1	7

Treatment response	Complete remission	5	13	18
	Partial remission	13	2	15
	No response	2	0	2

**Table 2:** Connexin 43 protein expression in relation to bone marrow (BM) involvement and grade of follicular lymphomas.

Cases	Connexin 43 immunoscore		Sum	Significance
	Low (0-1)	High (2-3)		*P*
BM involvement	5	13	18	No significance (0.699)
No BM involvement	3	12	15	

Grade (low: I-II)	5	19	24	No significance (0.651)
Grade (high: III)	3	6	9	
